# From pollution to reforestation: the hidden microbiome of *Alnus glutinosa* nodules over 30 years

**DOI:** 10.1038/s41598-025-07006-5

**Published:** 2025-07-02

**Authors:** Ryan Michael Thompson, Maria del Carmen Montero-Calasanz, David George, Edward M. Fox

**Affiliations:** 1 School of Natural and Environmental Sciences, Newcastle University, Newcastle upon Tyne, NE1 7RU UK; 2 IFAPA Las Torres-Andalusian Institute of Agricultural and Fisheries Research and Training, Junta de Andalucía, Cra. Sevilla-Cazalla, km 12.2. 41200, Alcalá del Río, Seville, Spain; 3 Department of Applied Sciences, Northumbria University, Newcastle upon Tyne, NE1 8ST UK

**Keywords:** Actinorhizal, Phytoremediation, *Frankiaceae*, Endosymbiosis, Pollutants, Microbial ecology, Microbial communities, Environmental microbiology

## Abstract

Actinorhizal plants, such as *Alnus glutinosa*, play a critical role in ecosystem restoration, particularly in metal-contaminated soils, yet their nodule microbiome remains largely unexplored beyond *Frankiaceae* endosymbionts. This study presents the first comprehensive analysis of *A. glutinosa* root nodules under heavy metal stress, focusing on a 30-year-old chronosequence planted upon opencast coal mine spoil. Microbial diversity analysis revealed that *A. glutinosa* nodules harbour a distinct and conserved microbiome, dominated by *Frankiaceae* but also enriched with plant growth-promoting bacteria such as *Bradyrhizobium*,* Mycobacterium*, and *Actinoplanes*. Additionally, despite similar beta diversity between the nodules and soil, significant compositional differences were observed, reinforcing the selective nature of the nodules. However, functional profiling indicated that metabolic pathways were largely shared between nodule and soil microbiomes. Overall, this study provides new insights into the resilience and specialisation of the *A. glutinosa* nodule microbiome and its potential role in bioremediation within heavy metal-contaminated environments.

**Supplementary Information:**

The online version contains supplementary material available at 10.1038/s41598-025-07006-5.

## Introduction

*Alnus glutinosa* is an actinorhizal plant species that together with their *Frankiaceae* endosymbionts, display a large potential in bioremediation, facilitating improvements to soil nutrients, revegetation of polluted sites and removal of soil pollutants^1^. Despite this potential, the root nodule community of *A*. *glutinosa* is highly understudied, aside from *Frankiaceae*, despite this niche being host to a range of other organisms^2–8^.

Currently only two published studies have directly investigated the microbial inhabitants of the *A. glutinosa* nodules. As expected, the majority of the retrieved bacterial species were *Frankiaceae*, with other taxonomic groups constituting minor proportions of the nodule^9^. In a later study conducted by McEwan et al.^10^, upon senescent nodules, a range of opportunists colonised the decaying nodules, with limited *Frankiaceae* present. As such it was assumed to not be representative of the nodule’s typical community.

More recent studies analysing the general root microbiome of *A. glutinosa*, including nodules, have reported limited recovery *Frankiaceae* and instead a range of other bacteria constituted the bulk of the root-associated community^11,12^. As noted by Thiem et al.^11^, this likely stems from the inclusion of bulk root material, as the nodules and absorptive roots harbour distinctly different bacterial distributions. However, none of these studies have examined heavy metal contaminated environments. Consequently, the diversity, community dynamics, and the potential bioremediation roles of the nodule inhabitants under such conditions remain unknown.

The Varteg Hill research site is situated upon opencast coal mine spoil in South Wales, United Kingdom (Supplementary Figure S1; Supplementary Table S1), with reclamation attempts dating back to 1963. Initial attempts at reclamation were relatively unsuccessful and the site was largely abandoned until 1990, when academics of Oxford Brookes University and volunteers initiated a restoration programme. This involved planting various plots with several tree species, including *A. glutinosa*^13^. This planting, done at various stages between 1991 and 2007, created a long term chronosequence which affords unique insight into how the nodule microbiome develops under heavy metal stress^14^ (https://cradlefornature.org.uk/).

This study explores the microbiome dynamics of the *A*. *glutinosa* nodule across the Varteg site chronosequence and represents the first analysis of root nodules of *A*. *glutinosa* under heavy metal polluted conditions. By uncovering how the nodule community responds to heavy metal stress, our findings provide critical insights into the microbial drivers of adaptation and resilience. Identifying key organisms involved in bioremediation could lay the foundation for developing targeted bioinoculants to enhance *A. glutinosa*-mediated phytoremediation, offering new strategies for ecosystem restoration in polluted environments.

## Methods

### Soil and root nodule sampling

Four *A*. *glutinosa* containing plots were sampled within the Varteg site on the 11th of August 2021. These consisted of the control plots of Svetla 3, Titania, Cariad and Mansi’s plot, aged 30, 27, 18 and 14 years old, respectively, at the time of sampling, planted according to Haigh et al.^15^, Plamping et al.^13^, Desai et al.^14^, Haigh et al.^16^, and Filcheva et al.^17^ (Supplementary Table S1).

Root nodules from three trees in each of the four plots were gathered by manual excavation using a trowel, with soil gathered from around the base of these trees. Due to the presence of rocks, tree roots and other natural debris, a set sampling distance from the trunk could not be used for each tree, so soil samples were taken as close to the tree as possible. Soil was collected at both 10 cm and 20 cm depths using an auger, with 3 cm of soil above and below these depths taken to ensure there was sufficient material for analysis. All individual soil and nodule samples were stored in separate plastic bags and kept in a cool box containing ice packs for transport to the laboratory, after which they were frozen (-20 °C) within 24 h of collection.

In addition to the selected plots, unplanted soil to the north of Mansi’s plot was also collected to act as a tree-free control (Supplementary Table S1). *A*. *glutinosa* root nodules of two trees at Saltwell park, and surrounding soil, were collected from Gateshead, United Kingdom (54°56’41.0"N 1°36’21.1"W and 54°56’40.2"N 1°36’17.6"W) on 31st March 2021 to act as an unpolluted control.

All plant material collected from Varteg and Saltwell park were collected with the relevant permissions sought. The sampled *A. glutinosa* was identified by R. M. Thompson based upon the leaf appearance (racquet shaped, leathery texture with slightly serrated edges), presence of purple-grey leaf buds and the presence of male and female catkins. In addition to the presence of root nodules when the roots were uncovered.

### Soil heavy metal and pH analysis

Soil pH was determined by suspending 5 g of soil in 12.5 ml of distilled water and shaken on an orbital shaker (KS 500 orbital shaker, Janke & Kunkel) for 10 min at 160 Hz. A pH probe was inserted into the interface of the soil and water, with the pH read once stable. Soil pH data was statistically analysed using Minitab (version 21.2.0.0). The distribution of the data was assessed using the multiple probability plot tool, after which the data was statistically analysed using a one-way ANOVA.

Heavy metal measurements of the soil samples were undertaken using X-ray fluorescence (XRF) analysis. The samples were sieved through a 2 mm sieve and homogenised to a fine and uniform distribution using a mortar and pestle. This soil was dried overnight and 4 g of soil was mixed with 1 g of CEREOX wax (Fluxana) using a mixer mill for 10 s at a frequency of 25 Hz (Retsch MM 200 model mixer mill). The samples were pressed with ten tonnes of force in a hydraulic press (Specac^®^, Atlas 15T manual hydraulic press) yielding a pellet for XRF analysis.

The pellets were analysed using an XEPOS XRF (Spectro Ametek) and the accompanying XRF Analyzer Pro software (version 3.9.5), with this equipment calibrated using reference material: BGS102, ERM CD200, SRM 2710, GBW07403, GBW07411, GBW07313, GBW07405 and GBW07406. From the XRF output, 15 metals were selected as the focus of the analysis. Cadmium, lead, mercury, silver, thallium, antimony and aluminium were chosen as they have no biological function and cause serious negative health effects^18^ (https://randd.defra.gov.uk/ProjectDetails?ProjectID=13317&FromS). Zinc, manganese, nickel, arsenic, chromium, cobalt and copper were selected, as while they are essential trace elements (or thought to play a biological role in the case of arsenic), they can be toxic when in excess (https://randd.defra.gov.uk/ProjectDetails?ProjectID=13317&FromS). Finally, uranium and vanadium were selected due to their slow loss from the soil which allows for their accumulation over time (https://randd.defra.gov.uk/ProjectDetails?ProjectID=13317&FromS).

The XRF output was statistically analysed using Minitab (version 21.2.0.0). The distribution of the data was assessed using a multiple probability plot with the data from the different plots separated into different groups. In the case of the cobalt, cadmium, antimony, mercury and uranium XRF outputs, some data points contained an “<” symbol as they were below the equipment detection limit. In such cases, the “<” symbol was removed and the data point treated as usual during the analysis. After confirming the soil data was normally distributed, the differences between soil metals were analysed using a one-way ANOVA.

### Nodule and soil sample genomic DNA extraction

A method based upon Lundberg et al.^19^, was utilised to surface sterilise the nodules. In brief, 150–250 mg (differing due to differences in amount of adhering soil and nodule size) of nodule material was cut from the nodule mass with scissors/tweezers sterilised with 70% ethanol. These nodules were washed of bulk soil by suspending the nodules in 1 ml of phosphate buffered saline (PBS) (8 g/L NaCl, 0.2 g/L KCl, 1.42 g/L Na_2_HPO_4_, 0.24 g/L KH_2_PO_4_, pH 7.4), and vortexing them at high speed for ten seconds (Vortex-genie 2, Scientific industries). This was repeated by transferring the nodules to fresh PBS until the PBS was free from sediment.

The nodules were transferred to a tube containing 1 ml of PBS and placed into a floating tube rack within an ultrasonic cleaner containing water (Clifton™). These tubes were sonicated on the “intense setting” for 5 min, with an alternating cycle of 30 s sonication and 30 s rest. After sonication, the nodules were removed from the PBS and manually homogenised with a sterile plastic pestle and transferred to a DNeasy power soil pro kit bead tube (Qiagen). The nodule material was further homogenised by vortexing the samples at maximum speed for ten minutes, followed by homogenisation using a FastPrep-24 classic homogeniser (MPbio) at 6 m/s for 40 s. After homogenisation, the DNA extraction was conducted following the manufacturer recommendations, with the eluted DNA being suspended in 50 µl of elution buffer. The eluted DNA and blanks were subject to nanodrop™ (ND-1000 spectrophotometer, Nanodrop) and Qubit 2.0 (dsDNA high sensitivity assay kit, Invitrogen) analyses.

To ensure the quality of amplified DNA amplicons, the DNA extracts and procedural blanks served as the basis of 16S rRNA PCRs consisting of: 1 µl each of the 27F (5’-AGAGTTTGATCMTGGCTCAG-3’) and 1492R (5’-TACGGYTACCTTGTTAYGACTT-3’) primers at a concentration of 10 µM, 12.5 µl master mix (2X MyFi mix, BioLine), 8.5 µl of H_2_O and 2 µl of DNA extract. PCR negative controls were prepared with these consisting of the above components, except the 2 µl of eluate was replaced with 2 µl of H_2_O. The PCRs were run with the following parameters: initial denaturation at 95 °C for 60 s, followed by 35 cycles of 95 °C for 30 s, 57.3 °C for 20 s and 72 °C for 45 s. These PCRs were concluded with a final elongation step at 72 °C for 120 s (TC-412 thermal cycler, Techne). The PCR product was analysed using gel electrophoresis (Power pack 300, Bio-Rad) with a 1% agarose gel and visualised using a Gel Doc™ EZ gel documentation system (Bio-Rad).

The extraction and analysis of soil DNA was conducted using the same process as the nodule extraction and analysis, except that 250 mg of soil was used in the extractions and the surface sterilisation step omitted. Extractions were conducted with a procedural blank and a PCR negative control was also prepared in a similar manner to that detailed above.

### 16S rRNA community gene sequencing and analysis

16S rRNA gene sequencing of nodule and soil DNA extracts was conducted by NUomics at Northumbria University, based upon the Earth Microbiome Project protocol with modified primers^20–22^. The 16S rRNA gene PCRs consisted of: 1X platinum hot start PCR master mix, 0.2 µM of each primer and 1 µl of template DNA (total volume 25 µl). Alongside the DNA extracts, NUomics added a positive (NuPos) and negative (NuNeg) control sample within the 96 well plate. These PCRs were conducted with the following parameters: initial denaturation at 94 °C for 3 min, followed by 35 cycles of 94 °C for 45 s, 50 °C for 60 s and 72 °C for 90 s. The PCRs were concluded with a final elongation step at 72 °C for 10 min.

The PCR products were normalised using a Quant-iT™ PicoGreen™ dsDNA assay kit (Invitrogen) following the manufacturer’s instructions and pooled per 96 well plate. Each pool was cleaned using 1:1 Ampure XP: Pooled amplicon, after which the DNA fragment size was determined using a BioAnalyzer (Agilent Technologies) and the concentration determined using a Qubit™ 1X dsDNA high sensitivity assay kit (Invitrogen). The PCR pools were combined in equimolar amounts to create a single 4 nM library, denatured using 0.2 N NaOH for five minutes and diluted to a final concentration of 5 pM, supplemented with 15% PhiX and loaded onto a MiSeq V2 500 cycle cartridge and sequenced.

The returned 16S rRNA gene reads from NUomics were processed using the Mothur pipeline within Galaxy Europe (https://usegalaxy.eu/)^23–32^, using the SILVA (version 138.1) alignment and taxonomy files. The SILVA files were downloaded from the Mothur wiki to ensure it was formatted correctly for use with the Mothur pipeline (https://mothur.org/wiki/silva_reference_files/).

Further analysis, was conducted upon the MicrobiomeAnalyst server using the SILVA taxonomy labels (https://www.microbiomeanalyst.ca/MicrobiomeAnalyst/), the consensus taxonomy output file and the share output file of Classify.OTU and Make.shared, respectively^33–35^. No data filtering was conducted using the MicrobiomeAnalyst server and the data was normalised using rarefaction to the minimum library size, due to its lower false discovery rate when analysing samples with large disparity in the number of reads^36^.

Analysis of the microbiome was conducted in three individual manners to analyse the data. The nodule and soil data were analysed together, followed by analysis of each of the nodule and soil datasets separately. This was done to compare differences between the soil and nodule data and to compare inter-plot differences between soil and nodule samples separately. From the normalised data a rarefaction curve and Goods coverage values were generated to determine the sequencing coverage. Alpha diversity was calculated at the feature level using the Chao1, observed, Shannon and Simpson diversity indices, with statistical analysis conducted using either a T-test or ANOVA. Beta diversity at the feature level was calculated via principal coordinate analysis (PCoA) using the Bray-Curtis index distance method and the PERMANOVA statistical test. A dendrogram was also created for all samples at the feature level using the Bray-Curtis index distance measure and the Ward clustering algorithm.

Linear discrimination analysis effect size (LEfSe) comparing the soil and nodule samples with FDR adjusted P-value cut-offs and a log LDA score of two was calculated at the genus level. This analysis however could not be conducted for the soil and nodule groups individually, due to the minimum group size requiring at least three samples and the park samples only having two samples. Instead, for the soil and nodule data the core microbiome was determined at the genus level, with sample prevalence and relative abundance being 20% and 0.01%, respectively. Community composition of the soil and nodules samples were visualised using the stacked bar/area plot at both the phylum and the genus level (displaying the top 25 most common genera).

### Greengenes annotation and PICRUSt analysis

To analyse the functional profile of the community the data was annotated using the Greengenes reference, raw data was again processed through the Mothur pipeline, but instead utilising the May 2013 Greengenes reference taxonomy and alignment file formatted for the Mothur pipeline (downloaded from https://mothur.org/wiki/greengenes-formatted_databases/). The output files were uploaded to MicrobiomeAnalyst and analysed using the phylogenetic investigation of communities by reconstruction of unobserved states (PICRUSt) tool^37–40^. The KEGG orthology (KO) table and metadata files were analysed under the shotgun data profiling section of MicrobiomAnalyst, with the gene ID type selected as “KEGG orthology (KO)”.

From the resulting files, various analyses were conducted which included PCoA, pattern search and functional diversity profiling. Functional diversity profiling was conducted based upon the COG functional category, with the abundance calculated using the total hits metric which is the sum of all hits belonging to each category (where KOs belonging to multiple groups are counted multiple times). Principal component analysis of the functional pathway was conducted by grouping the samples based upon sample type. Finally, pattern search was conducted using Pearson r distance measure to measure differences in KOs between the soil and nodule sample groups.

## Result and discussion

### Soil pH

Soil pH analysis demonstrated the mean soil pH of the planted Varteg plots ranged from 4.99 to 5.86, with the unplanted plot having a mean pH of 6.11 (Table 1). Previous analysis of the Varteg soil pH indicated that the unplanted soil pH varies from 5.2 to 5.7 with “hotspots” having a pH as low as 3.8^16,41^. The planted sites of this investigation generally fall within the expected pH range; however, the unplanted soil exhibits higher-than-anticipated values. This discrepancy may stem from the heterogeneous nature of the Varteg site. Additionally, since previous pH measurements of unplanted plots were recorded decades ago, ongoing bioremediation processes may have influenced the surrounding unplanted areas over time.

**Table 1:** Mean XRF values and mean soil pH values of the Varteg and saltwell park sample, alongside a wax blank, with standard deviation noted in brackets. Mean soil metals values were calculated using a two-way ANOVA with significant differences (0.05) denoted by a pairwise Tukey test. Mean soil pH values were calculated using a one-way ANOVA with significant differences (0.05) denoted by a pairwise Tukey test (S = 0.236259, R^2^ = 90.13%). All soil metal data was normally distributed aside for the Cobalt data points, the cadmium data points of svetla, unplanted, mansi’s and titania, alongside the mercury data points of Svetla and titania. These were unable to be calculated as the values were the same for each replicate of these plots, due to the values of these being below the analysis detection limit.

	mg/kg of element in soil																
Sample	V	Cr	MnO	Co	Ni	Cu	Zn	As	Ag	Cd	Sb	Hg	Tl	Pb	U	Al_2_O_3_	pH
Wax blank	< 0.1	0.13	1.7	< 0.1	6.8	2.3	0.75	< 0.1	< 0.5	< 1.0	7	< 1.3	< 0.1	< 0.1	< 0.1	< 0.00070	-
Park sample	169.8^b^ (29.3)	677^a^ (584)	1923^a^ (611)	-	38.55^a^ (5.44)	85.1^a^ (44.1)	200.9^a^ (30.7)	17.40^a^ (5.94)	0.35^a^ (0.20)	0.41^a^ (0.43)	1.65^a^ (0.63)	0.35^a^ (0.07)	1.60^a^ (0.56)	128.4^a^ (48.9)	0.75^c^ (0.49)	10.24^b^ (1.10)	6.69^a^ (0.009)
Unplanted	260.4^a^ (28.1)	469^a^ (205)	1318^a^ (335)	-	49.93^a^ (0.05)	34.80^b^ (4.33)	93.23^bc^ (4.42)	16.57^a^ (5.94)	0.44^a^ (0.05)	0.1^a^ (0.00)	1.06^a^ (0.89)	0.33^a^ (0.05)	1.33^a^ (0.20)	35.03^b^ (7.44)	3.23^a^ (0.15)	19.34^a^ (2.33)	6.11^ab^ (0.05)
Mansi’s	274.7^a^ (24.0)	643^a^ (262)	880^a^ (226)	-	48.23^a^ (7.13)	35.33^b^ (5.92)	76.93^bc^ (15.57)	9.20^a^ (1.20)	0.49^a^ (0.07)	0.1^a^ (0.00)	0.43^a^ (0.05)	0.33^a^ (0.05)	1.20^a^ (0.20)	31.13^b^ (4.01)	3.33^a^ (0.23)	21.54^a^ (0.43)	5.86^bc^ (0.12)
Cariad	271.2^a^ (19.4)	582.6^a^ (21.2)	1607^a^ (369)	-	48.83^a^ (1.93)	37.47^b^ (2.95)	82.77^bc^ (11.96)	11.73^a^ (1.38)	0.54^a^ (0.11)	0.13^a^ (0.05)	0.36^a^ (0.05)	0.33^a^ (0.05)	1.43^a^ (0.20)	56.90^b^ (15.45)	2.97^ab^ (0.51)	19.37^a^ (2.34)	5.32^cd^ (0.20)
Titania	296.13^a^ (14.30)	539.2^a^ (106.4)	775^a^ (482)	-	43.20^a^ (1.87)	36.06^b^ (1.45)	72.50^c^ (9.51)	13.87^a^ (2.78)	0.44^a^ (0.11)	0.1^a^ (0.00)	0.40^a^ (0.10)	0.30^a^ (0.00)	1.40^a^ (0.10)	58.27^b^ (8.88)	2.13^b^ (0.30)	20.80^a^ (1.86)	4.99^d^ (0.46)
Svetla	249.23^a^ (13.56)	393^a^ (206)	929^a^ (204)	-	50.33^a^ (5.00)	40.33^b^ (3.54)	112.63^b^ (10.77)	12.46^a^ (0.60)	0.61^a^ (0.03)	0.1^a^ (0.00)	0.36^a^ (0.20)	0.40^a^ (0.00)	1.13^a^ (0.15)	45.63^b^ (11.56)	3.36^a^ (0.23)	20.02^a^ (0.30)	5.06^d^ (0.16)

Soil pH analysis demonstrated that the mean soil pH of the Varteg plots decreased with plantation age, with the unplanted site having the highest mean pH (Table 1). There is limited data available within the literature regarding the Varteg site to make comparisons. However, other studies utilising *Alnus*, such as Kuznetsova et al.^42^, and Callender et al.^43^, already reported a decrease in soil pH. This decrease in soil pH likely stems from *Alnus-Frankiaceae* nitrogen fixation which increases the pool of ammonium and nitrates within the soil which can lead to lower soil pH^44,45^. Furthermore, organic acids produced by both plants and microbes, to solubilise nutrients, may also contribute to lower soil pH^46^.

### Soil heavy metals

#### Saltwell park soil heavy metals compared to Varteg

Initially it was expected that metals would be higher in the Varteg samples compared to Saltwell park. However, only six of the 15 analysed metals were significantly different between the two sites (Table 1). This is supported by data from the UK Soil Observatory (https://www.ukso.org/static-maps/advanced-soil-geochemical-atlas-of-england-and-wales.html) where Saltwell exhibited higher or comparable mean metal values to Varteg, except in the case of arsenic, cadmium and magnesium (mercury was not considered by the UK soil observatory).

Such metal pollution in Saltwell park may stem from historic pollution linked to mining, industrial, and heavy engineering. Similar metal accumulation has been documented in other areas of Tyne and Wear, where Saltwell park is situated; in the case of a nearby site, soil contamination was so severe that pollution from a waste incinerator was indistinguishable from the background metal levels^47,48^. This likely explains why Saltwell Park exhibits higher metal concentrations than most UK soils and why it may not serve as a metal-free control site as originally intended, though it remains valuable for comparative analyses.

#### Varteg inter-plot differences in heavy metals

Regarding differences between the Varteg plots, only zinc and uranium showed significant differences, but without any clear pattern to suggest why some plots had higher amounts of metals relative to others (Table 1). This broad lack of significant differences and discernible patterns was unexpected, as phytoextraction of soil metals by *Alnus* would be expected to lower soil metals in proportion to the plot age, as suggested by Desai et al.^14^. Nevertheless, it was noteworthy that soil metal levels of the planted plots in this study were generally lower than those presented in Desai et al.^14^ from samples collected in 2010. This suggests that bioremediation may be continuing, with soil metal levels declining over time.

### Nodule community alpha diversity analysis

When considering alpha diversity of samples taken from the Varteg site, rarefaction curves of all samples seemed to be reaching saturation, evident by the curves beginning to plateau (Supplementary Figure S2). This is supported by the Goods coverage values obtained, with the lowest of these being 90.08% (Supplementary Table S2), indicating adequate sequencing coverage of the samples.

**Fig. 1:**
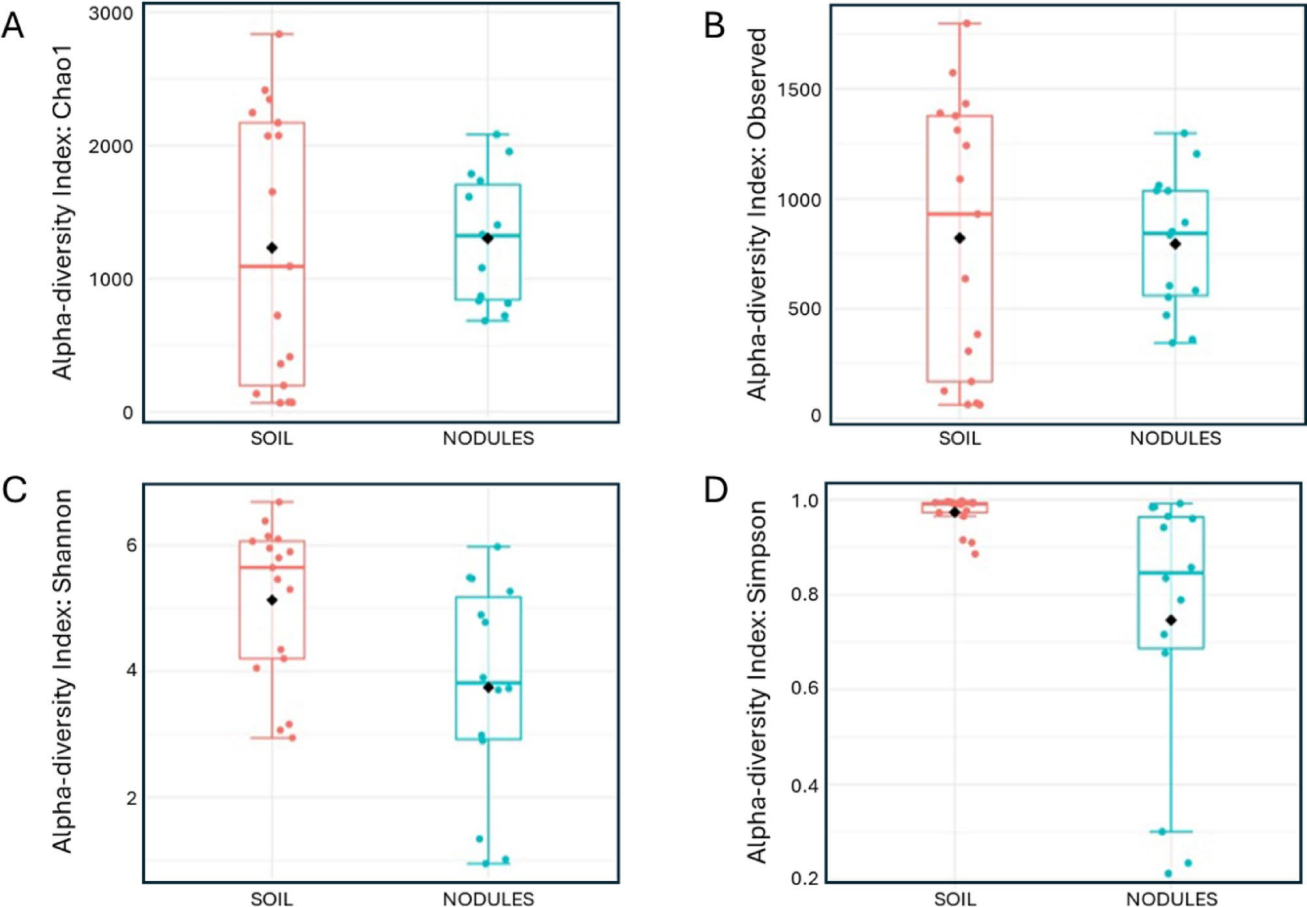
Alpha diversity indices of the soil and nodule samples at the OTU level. A, Chao1 (p-value: 0.8053; [T-test] statistic: -0.24928); B, Observed OTU (p-value: 0.87917; [T-test] statistic: 0.15362); C, Shannon index (p-value: 0.018484; [T-test] statistic: 2.5345);D, Simpson (p-value: 0.011362; [T-test] statistic: 2.9346). Each box plot represents the diversity distribution of the samples in each group.

Alpha diversity of the soil and nodules were compared using the Chao1, observed OTUs, Shannon and Simpson indices, which yielded P-values of 0.8053, 0.87917, 0.018484 and 0.011362, respectively (Fig. 1). The P-values of the Chao1 and observed alpha diversity indices suggest no significant differences in alpha diversity between nodule and soil samples. However, these metrics do not account for species evenness. In contrast, the Shannon and Simpson indices, which incorporate species evenness, reveal significant differences in alpha diversity between soil and nodules.

These differences in evenness are supported by the nodules being more enriched with *Frankiaceae* compared to soil. In addition, the nodule is a more specialised niche compared to the soil^49 ^so it is expected that a lower amount of non-*Frankiaceae* species can effectively colonise this niche. Similar results have been reported for *Casuarina glauca*, with the nodules displaying reduced alpha diversity compared to the surrounding soil^50^. Furthermore, non-actinorhizal plants have also exhibited lower Shannon and InvSimpson index values in the root interior compared to the soil and root exterior^51^.

When comparing the alpha diversity of nodules samples collected from differing Varteg plots and those derived from Saltwell park, the Chao1, observed, Shannon and Simpson indices yielded P-values of 0.72713, 0.68615, 0.78122, and 0.88352, respectively, indicating no significant differences between these sites (Supplementary Figure S3). This lack of variation may be due to the long-established plantations, allowing the development of conserved microbial communities across plots. While other studies have reported differences in actinorhizal nodule alpha diversity, those cases involved sites with varying climate conditions, which could influence microbiome composition^50^. In contrast, the present study primarily sampled from a relatively close geographic location with similar climates, likely reducing environmental variability as a factor.

### Nodule beta diversity compared to soil

**Fig. 2:**
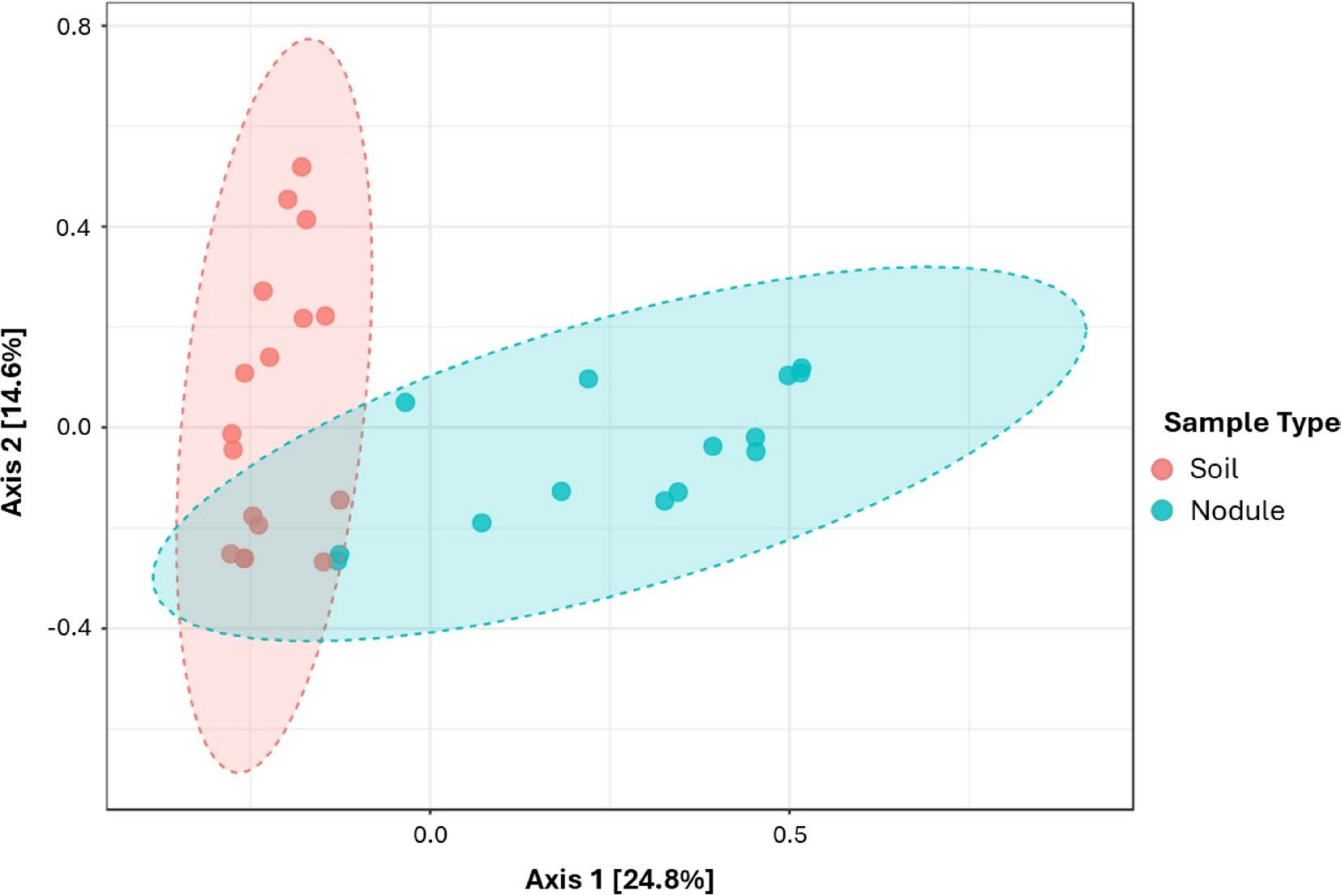
Beta diversity analysis conducted for soil and nodule samples represented using two-dimensional principal component analysis calculated using Bray distance. [PERMANOVA] F-value: 6.9083; R-squared: 0.19239; p-value: 0.001.

In terms of beta-diversity, nodule and soil samples were largely similar, with only slight dissimilarity as reflected by the Bray-Curtis R^2^ value of 0.19239 (*P* = 0.001) (Fig. 2). This similarity likely arises from soil microorganisms colonising the nodule, a phenomenon previously observed in *Alnus* nodules by McEwan et al.^10^. The minor beta-diversity variation between nodules and soil may be attributed to certain organisms struggling to establish and persist within the nodule environment, which is more specialised, microaerophilic, and characterised by high interspecific competition^49^.

The beta-diversity pattern observed in this study align to some extent with those reported by Ghodhbane-Gtari et al.^50^, where *C. glauca* nodules differed from the surrounding soil, showing comparable weighted and unweighted UniFrac results to the Bray-Curtis index used in this study. However, the nodule-soil differences in Ghodhbane-Gtari et al.^50^, appeared more pronounced when plotted on a PCoA. Other investigations, such as Chen et al.^51^, have reported distinct microbial communities between soil and root interiors, with greater diversity observed inside the roots. However, that particular study only considered the most dominant taxa, potentially underestimating the full extent of microbial diversity within these niches.

Beta-diversity of nodule samples alone revealed no significant differences observed between nodules collected from differing plots (R^2^ = 0.37616, *P* = 0.197) (Supplementary Figure S4). Nevertheless, while some variation exists, the R^2^ value does not indicate complete dissimilarity. Akin to the alpha diversity, this lack of significant beta diversity differences is likely due to the long-term stability of the planted sites, allowing the establishment of a conserved microbial community^52,53^. Additionally, *Frankiaceae*, which are highly specialised for the nodule niche, dominate the microbiome, limiting colonisation by other microorganisms and thereby reducing beta diversity. The specialised nature of the nodule environment compared to soil further reinforces this pattern^49^. These findings suggest that a conserved microbial community has developed not only within the Varteg nodules but also between Varteg and Saltwell Park, indicating the potential for conserved nodule communities across different sites.

### Community structure of the nodules

**Fig. 3:**
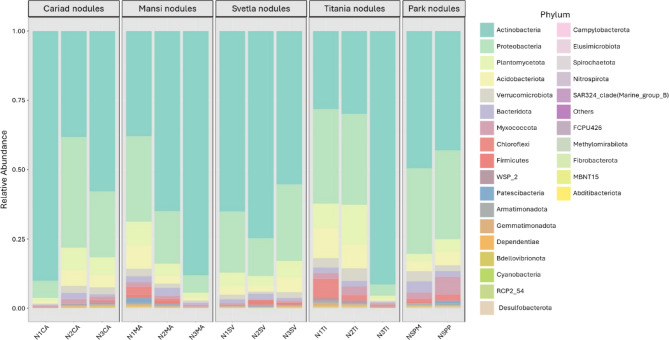
Percentage abundance of taxonomic groups at the phylum level for the nodule communities. The Y-axis indicates the relative (%) abundance with the samples displayed on the X-axis, grouped by plots.

At the phylum level, the nodule microbiome is predominantly composed of *Actinobacteriota*, with *Proteobacteria*,* Planctomycetota* and *Acidobacteriota* also present in smaller but significant proportions, alongside a diverse array of other phyla in minor abundances (Fig. 3).

**Fig. 4:**
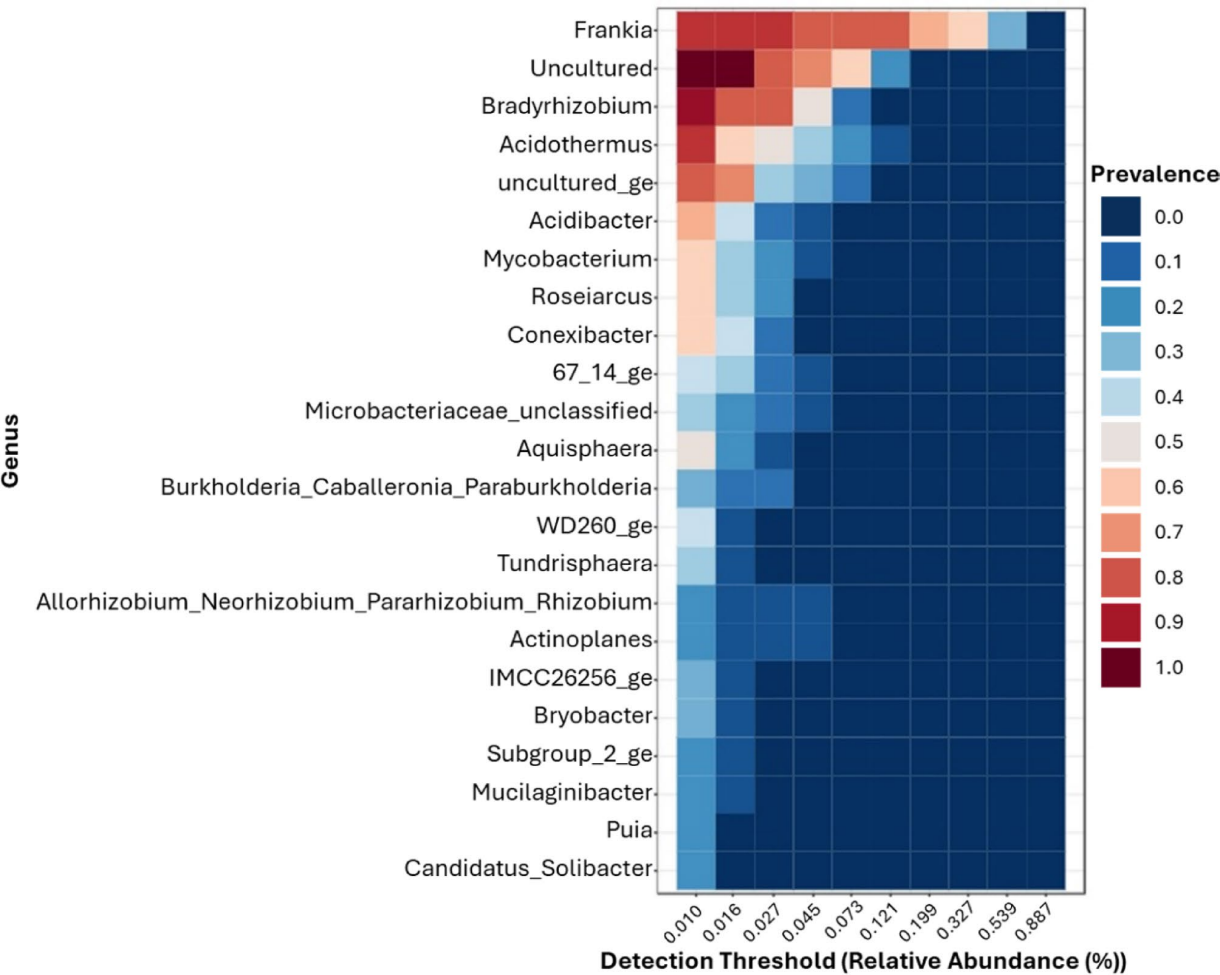
Genus level core microbiome composed of all nodule samples in this investigation. Prevalence relates to the number of samples in which the OTU is located at the relative abundance threshold specified upon the X-axis.

*Frankiaceae* overwhelmingly dominate the nodule microbiome compared to the surrounding soil, as they are the primary drivers of nodule formation (Fig. 4; Supplementary Figure S5). Additionally, the core microbiome includes other well-known plant growth-promoting (PGP) bacteria, such as *Bradyrhizobium*^54 ^as well as members of the *Rhizobiaceae*^55–59^ and *Burkholderiaceae* families^60–63^. *Bradyrhizobium* is the third largest group within the nodule core microbiome, while not being a major part of the soil microbiome, lending credence to the notion that the nodule is a selective environment. *Bradyrhizobium* representatives are well-known PGP bacteria that naturally inhabit soil but also the root nodules of certain legume species. They exhibit a range of PGP activities, most notably nitrogen fixation, phosphate solubilisation, and siderophore production^54^. These functions have been widely reported to enhance legume growth, which in turn may support more effective bioremediation^64,65^ by promoting faster plant development.

*Mycobacterium* was also identified within the core nodule microbiome and has previously been recovered in culture-dependent studies of nodule-associated bacteria^6^. Although less frequently associated with plant growth promotion than the previously mentioned groups, *Mycobacterium* have demonstrated PGP activity^66–68^. This was even evident under heavy metal stress, with *Mycobacterium* sp. ACC14 inoculation significantly increasing several plant growth metrics of *Brassica napus* under cadmium stress^67^. Suggesting a potential similar PGP role within the nodules akin to *Bradyrhizobium.*

Additionally, *Actinoplanes*, known for their antifungal properties and ability to enhance plant growth, were present in the core microbiome, indicating a possible functional role^69–71^. Recently, Hagagy and AbdElgawad^72^, demonstrated that *Actinoplanes* sp. PGPB5, isolated from heavy metal polluted soil, was able to improve the tolerance of wheat plants to thallium stress. This effect was attributed to a reduction in oxidative stress, as inoculated wheat plants showed upregulation of a range of genes involved in antioxidant and detoxification activities. These findings suggest that *Actinoplanes* may play a beneficial role within nodules under stressful environmental conditions. Finally, *Mucilaginibacter*, a well-characterised genus with documented PGP activity^73–75 ^was also found within the core nodule microbiome. In addition to PGP activity, *Mucilaginibacter* have been isolated from heavy metal polluted environments and are known to harbour heavy metal resistance genes which may aid in metal modification and sequestration^76–78^. Notably, the extrapolymeric substance of *Mucilaginibacter rubeus* P2, which was upregulated under metal stress, showed a high degree of bio-adsorption of a range of metals^79^. Therefore, the production of such substances may aid in bioremediation via metal binding and immmobilisation; moreoer, the toxicity of some metals, such as zinc and copper, was shown to decrease via complexing with the extrapolymeric substance^79^.

Aside from these groups, a significant portion of the nodule microbiome is composed of uncultivated taxa and underexplored genera, including *Acidothermus*, *Acidibacter* and *Roseiarcus*, amongst others (Fig. 4; Supplementary Figure S5). This highlights the need for culture-dependent studies to isolate and characterise these microorganisms, shedding light on their functional role within the nodule environment.

In comparison to the soil microbiome, several taxa such as *Aquisphaera*, *Conexibacter* and *Roseiarcus*, amongst others, were present in both niches, likely due to soil-derived colonisation of the nodules. However, their relative abundance differed between the two microbiomes. For instance, *Acidibacter* ranked as the sixth most abundant member of the nodule core microbiome but only the 28th most abundant in the soil core microbiome (Figs. 4; Supplementary Figure S5, S6). These distributional differences highlight the distinct nature of the nodule microbiome in contrast to its surrounding soil environment.

A range of groups, such as *Pseudomonas*, *Haliangium*, *Candidatus Udaeobacter*, *Pseudolabrys* and *Pajaroellobacter*, were identified within the soil core microbiome whilst being absent from the nodule core microbiome (Figs. 4; Supplementary Figure S6). Likewise, members of the *Frankiaceae*, *Rhizobiaceae* and *Burkholderiaceae* families, alongside members of the genera *Bradyrhizobium*, *Mycobacterium*, *Tundrisphaera*, *Actinoplanes*, *Mucilaginibacter* and *Puia*m, were exclusive to the nodule core microbiome and absent from the soil. This distinct microbial composition further reinforces the idea that the nodule harbors a specialised core microbiome that differs from the surrounding soil.

In comparison to the literature, the microbiome detailed by McEwan et al.^10^, was dominated by opportunistic microorganisms, most likely due to the nodules being in a senescent state. As a result, differences between their findings and the present study are expected. Focusing on active nodules, McEwan et al.^9^, reported that the majority of the nodule endophyte 16S rRNA gene sequences were *Frankiaceae* in origin, with some other species present within the nodules. This aligns broadly with the current study, where *Frankiaceae* also dominated the nodule microbiome in most cases. However, our findings suggest a greater diversity of non-*Frankiaceae* species within the nodules compared to those reported by McEwan et al.^9^, highlighting potential variations in microbial composition across different environmental conditions or sampling approaches.

Thiem et al.^11,12^ analysed *A. glutinosa*-associated communities and reported a lack of *Frankiaceae*, in contrast to both the present study and the findings by McEwan et al. (2015)^9^. However, this discrepancy is expected, as their studies focused on general root-associated microbiomes rather than nodule-specific communities, where *Frankiaceae* is predominantly localised. Additionally, only *Rhizobium*, *Granulicella* and *Bradyrhizobium* were shared between Thiem et al.^11^ and the current study. While these groups constituted relatively minor fractions of the community in Thiem et al.^11 ^*Bradyrhizobium* represented a more substantial portion of the core microbiome in the nodules of the nodules analysed here (Fig. 4; Supplementary Figure S5), further underscoring the distinct composition of nodule-associated microbial communities.

The root material analysed by Thiem et al.^12^, comprised a diverse range of genera, with most present at abundances below 2%, except for *Rhizomicrobium*, *Streptomyces* and *Acidothermus*. Notably, *Rhizomicrobium* was absent from the core microbiome of nodules in the present study, while *Streptomyces* only constituted a minor fraction of the nodule community (Fig. 4; Supplementary Figure S5). In contrast, *Acidothermus* represented a larger portion of the nodule microbiome in this investigation (Fig. 4). Collectively, these results demonstrate the potential differences of *Alnus* nodules compared to general root-associated communities, emphasising the specialised nature of the nodule microbiome.

### Community functionality profiling of nodules compared to soil

**Fig. 5:**
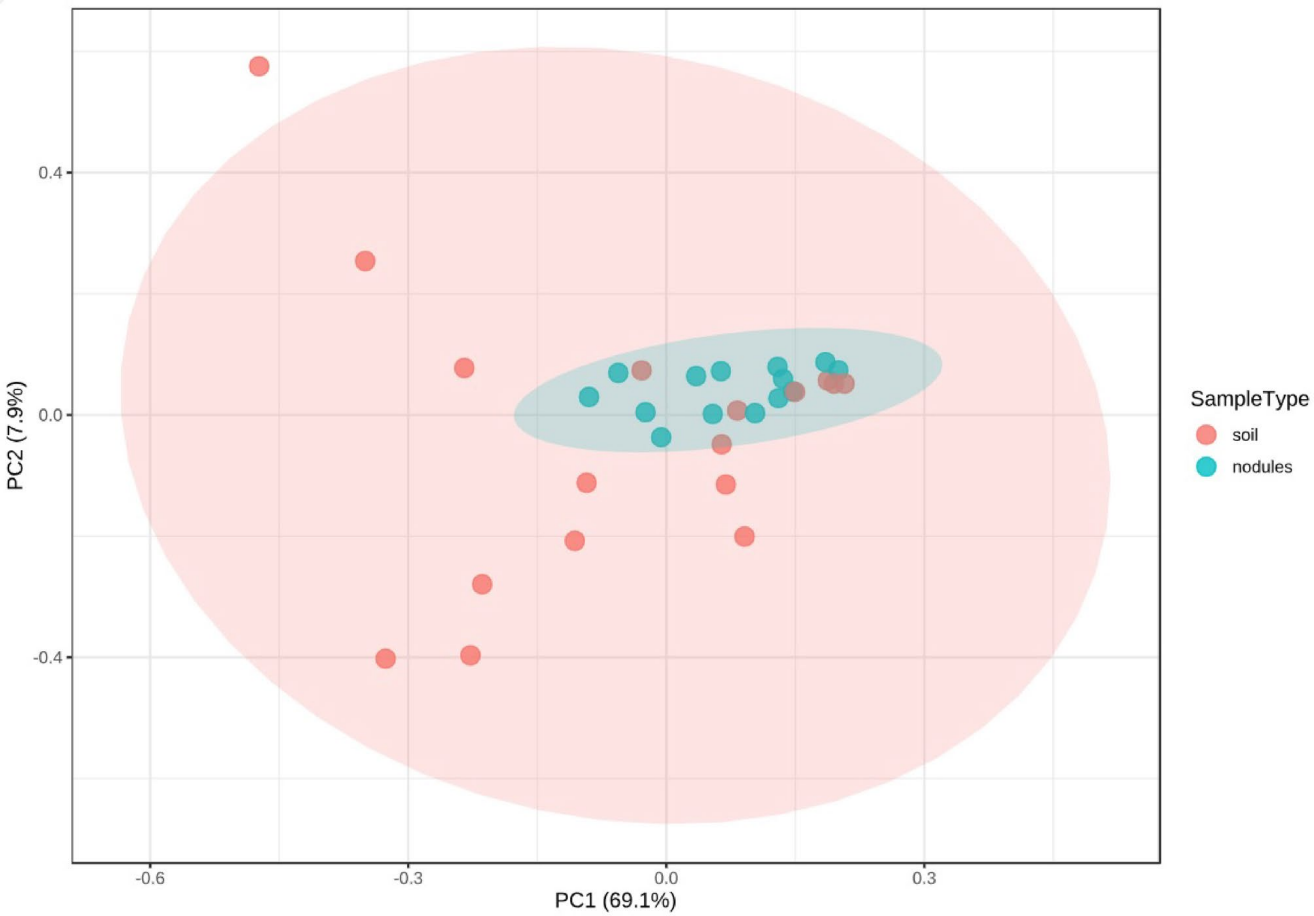
Principal component analysis conducted upon the samples based upon the KEGG orthologs (KO) present^38–40^. Soil samples are noted in red and nodule samples noted in blue.

Functional predictions based on COG categories revealed a broadly similar distribution between nodule and soil samples (Supplementary Figure S7). Likewise, principal component analysis of KEGG pathways showed that all pathways identified in the nodules are also present in the surrounding soil (Fig. 5). This functional prediction similarity aligns with the beta diversity data, as the comparable microbial composition between sample types suggests a corresponding overlap in functional potential. This is reflected in Ezeokoli et al.^52^, who reported that an 18-year old reclaimed site exhibited a functional profile similar to that of an unpolluted control, likely due to similarities in beta diversity. In addition, other studies have noted that root interiors tend to harbour lower functional diversity compared to the root exterior and surrounding soil^51^ a pattern in line with the observations in this study.

Pattern search was employed to identify significant differences in the KO groups between soil and nodules samples. However, only soil samples exhibited significant differences in KO groups, which align with the principal component analysis showing that all KEGG pathways present in the nodules are also found in the soil (Fig. 5). The 25 most distinct differences largely contained KEGG pathways unrelated to PGP and stress tolerance. Instead, these genes likely belong to metabolic pathways that are either absent from the nodules or more prevalent in the soil (e.g. K08305, K00833, K13942, K03763, K04518, K05281, K00378, K01935, K08096, K05979, K01906, K06133, K14138, K08481, K12214 and K07499). Additionally, K07105 and K06992 correspond to uncharacterised proteins, making their functional role within these microbiomes uncertain. Some KO groups related to two-component signalling systems were identified, including LuxR family (K13040 and K13041) and NarL family (K07689), but their specific functions remain unclear. Interestingly, K02915 was enriched in the soil, which was unexpected given that it encodes the large subunit ribosomal protein L34e, found in humans and eukaryotes. Since eukaryotic gene sequences were removed during 16S gene rRNA annotation, its presence raises questions about potential annotation artefacts or horizontal gene transfer events.

KO groups related to universal stress protein A (K06149) and multidrug resistance proteins (K03297, K08162) were significantly more abundant in the soil, with these plausibly contributing to metal tolerance. Initially, it was anticipated that certain pathways, such as nitrogen fixation, would be more enriched in the nodules compared to the soil, given the high abundance of nitrogen-fixing *Frankiaceae* in the former. However, the principal component analysis did not reflect this enrichment, possibly because other diazotrophs in the soil also contribute to nitrogen fixation.

## Conclusion

In conclusion, this investigation represents the first comprehensive study of actinorhizal root nodules under heavy metal stress. In terms of soils characteristics, pH decreased with plot age in the Varteg site, likely influenced by plant activity, particularly nitrogen fixation. However, no clear trends were observed in soil metal concentrations across the Varteg plots, and metal levels in Saltwell Park samples were generally comparable to those in Varteg. The bacterial community within the nodules largely mirrored that of the surrounding soil microbiome. However, there was a higher abundance of *Frankiaceae* in the nodule core microbiome compared to the soil. Likewise, there was differences evident in the distribution of bacterial groups between the nodule and soil core microbiomes. Suggesting that, even after long-term plantation, the nodule develops a distinct microbial community, likely due selective pressures imposed by this niche.

However, many of the microbial constituents of *A. glutinosa* remain largely understudied. Future research should focus upon culture-based isolation combined with whole-metagenome sequencing to further characterise these communities. Such studies would provide deeper insights into the functional roles of these microbes, particularly in biochemical processes such as bioremediation.

## Electronic supplementary material

Below is the link to the electronic supplementary material.

Supplementary Material 1

## Data Availability

The amplicon sequence data was deposited within NCBI under the bioproject accession number PRJNA1195611, with the individual SRA datasets deposited between the accession numbers SRX27024451 - SRX27024483.
