# Supplementary material for: From pollution to reforestation: the hidden microbiome of Alnus glutinosa nodules over 30 years
Source: Sci Rep. 2025 Jul 2;15:23373. doi: 10.1038/s41598-025-07006-5 (PMC12222880; doi:10.1038/s41598-025-07006-5)
Supplement: Supplementary file 1 — Supplementary Material 1 [file 41598_2025_7006_MOESM1_ESM.docx]

**From pollution to reforestation: the hidden microbiome of *Alnus glutinosa* nodules over 30 years**

**Supplementary Table S1**. Information regarding the sampled Varteg plots. This information includes: the sites name, the planting date, the planting method and the fertiliser application that was administered at the time of planting. Spent mushroom compost (SMC) composed of NPK+Mg 0.24:0.06:0.31+0.04, organic municipal green waste compost (GWC).

| **Plot** | **Planting year** | **Planting method** | **Fertiliser application** | **Trees and soil GPS location (latitude, longitude)** |
| --- | --- | --- | --- | --- |
| Svetla 3 | 1991 | Trench | 1 kg SMC per stem | 51°44'41.445"N 3°4'49.069"W  51°44'40.981"N 3°4'49.324"W  51°44'41.307"N 3°4'49.217"W |
| Titania | 1994 | Trench | 1 kg SMC per stem | 51°44'42.872"N 3°4'44.939"W  51°44'42.923"N 3°4'44.704"W  51°44'42.885"N 3°4'44.726"W |
| Cariad | 2003 | Trench | 0.5 kg SMC per stem | 51°44'43.963"N 3°4'48.265"W  51°44'44.054"N 3°4'48.384"W  51°44'44.026"N 3°4'48.414"W |
| Mansi’s plot | 2007 | Notch | 1 kg GWC per stem | 51°44'45.858"N 3°4'39.472"W  51°44'45.612"N 3°4'39.235"W  51°44'45.799"N 3°4'39.284"W |
| Unplanted soil | N/A | N/A | N/A | 51°44'45.924"N 3°4'40.123"W  51°44'45.841"N 3°4'40.333"W  51°44'46.222"N 3°4'40.348"W |

**Supplementary Table S2.** Goods coverage statistics calculated during generation of rarefaction curves of the soil and nodule samples.

| **Samples** | **Goods coverage** | **Goods coverage** |
| --- | --- | --- |
|  | **Soil** | **Nodules** |
| Saltwell park boathouse sample | 91.3925303 | 93.7670047 |
| Saltwell park playpark sample | 90.08162256 | 93.98961167 |
| Cariad sample 1 | 92.1221865 | 97.39055157 |
| Cariad sample 2 | 92.32005936 | 95.22631709 |
| Cariad sample 3 | 92.76527331 | 95.04081128 |
| Mansi’s sample 1 | 99.41874845 | 93.04971556 |
| Mansi’s sample 2 | 97.22977987 | 94.68216671 |
| Mansi’s sample 3 | 99.91343062 | 96.80930002 |
| Svetla sample 1 | 98.56542172 | 96.26514964 |
| Svetla sample 2 | 99.22087559 | 96.83403413 |
| Svetla sample 3 | 94.3606233 | 97.01953995 |
| Titania sample 1 | 99.62898837 | 94.27405392 |
| Titania sample 2 | 99.88869651 | 92.83947564 |
| Titania sample 3 | 92.72817215 | 97.51422211 |
| Varteg unplanted 1 | 99.8392283 | - |
| Varteg unplanted 2 | 99.88869651 | - |
| Varteg unplanted 3 | 92.2829582 | - |


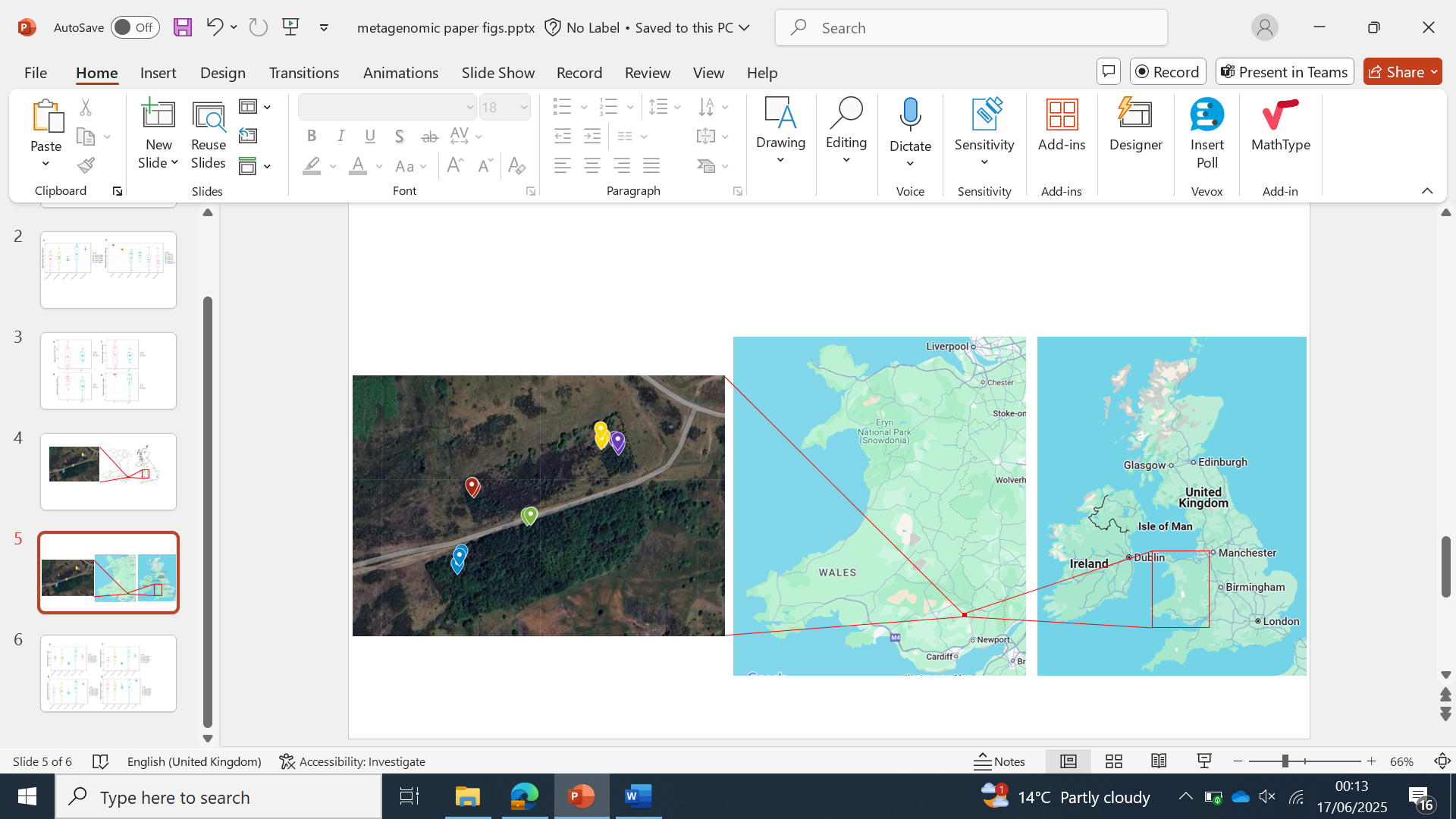
**Supplementary figure** **S1**. The approximate location of the sampled Varteg site in Wales and the location of Wales relative to the United Kingdom (map data: Google, ©2025). On the satellite site image, the blue markers indicate the Svetla three samples, green markers indicate the Titania samples, red markers indicate the Cariad samples, purple markers indicate the Mansi’s plot samples and the yellow markers indicate the location of the unplanted samples. The recorded GPS locations can be found in Supplementary Table 1.


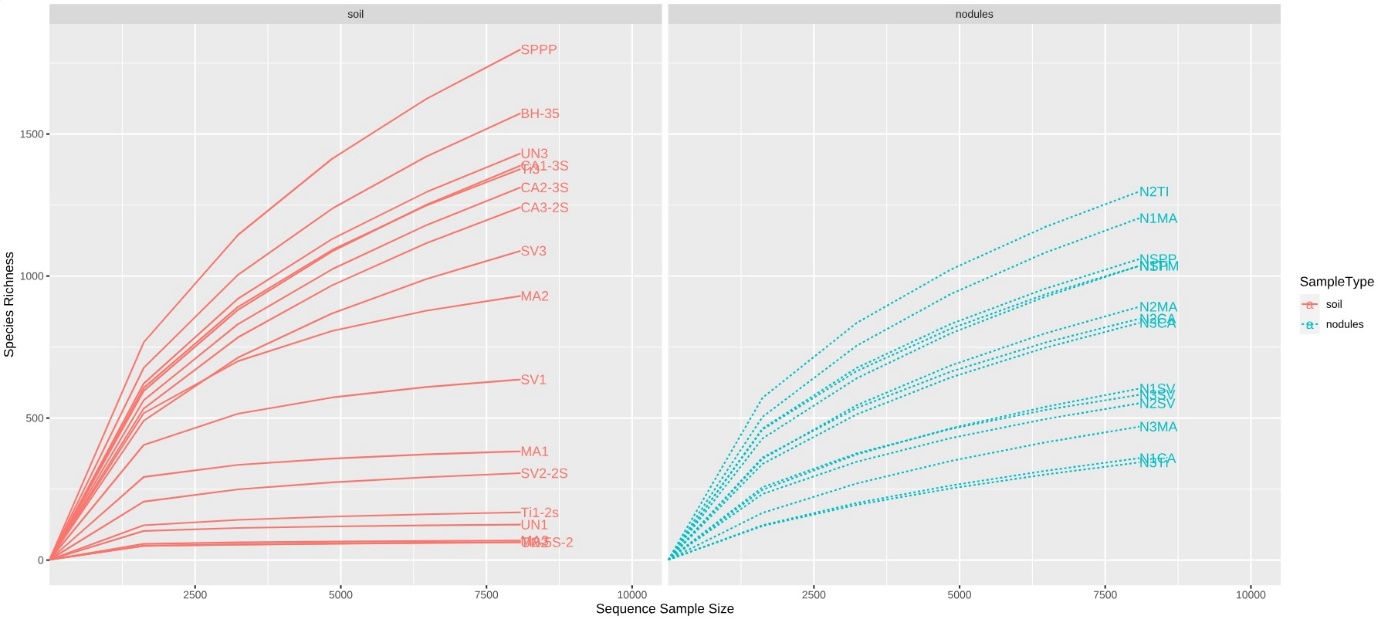
**Supplementary Figure S2**. Rarefaction curves of all samples for both the soil and nodule, generated using the MicrobiomeAnalyst webserver. The X-axis details the sequence sample size and the Y-axis details the sample richness.


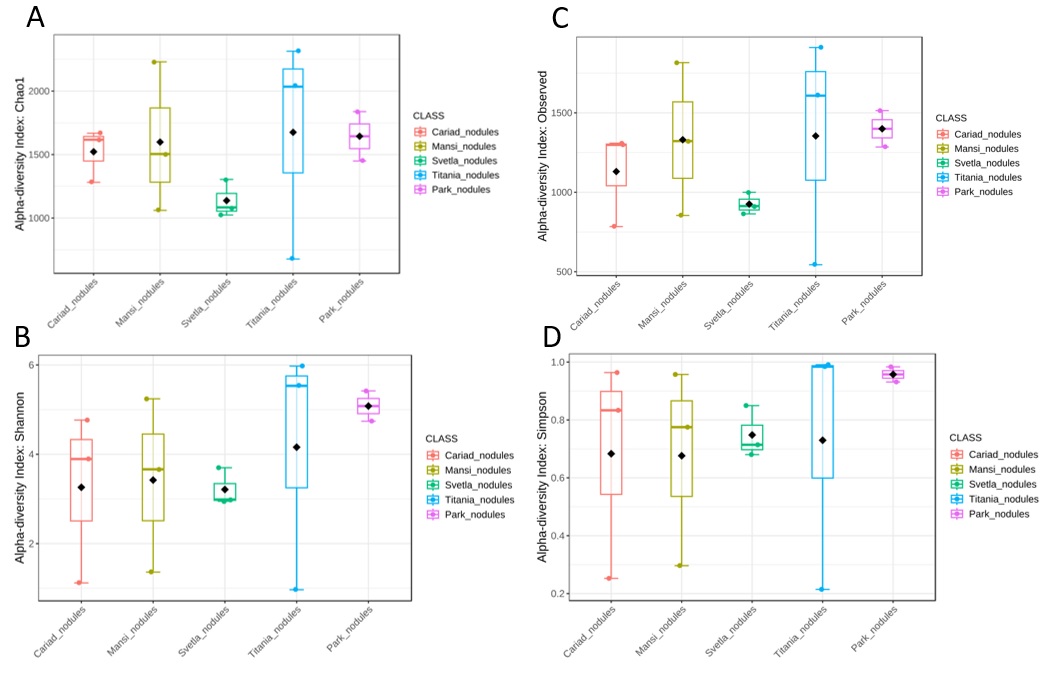
**Supplementary Figure S3.** Alpha diversity index of nodule samples at the OTU level for: A, Chao1 (p-value 0.72713; [ANOVA] F-value: 0.5149); B, observed OTU (p-value: 0.68615; [ANOVA] F-value: 0.57796); C, Shannon index (p-value: 0.78122; [ANOVA] F-value: 0.43395); D, Simpson (p-value: 0.88352; [ANOVA] F-value: 0.28026). Each box plot represents the diversity distribution of the samples in each group.


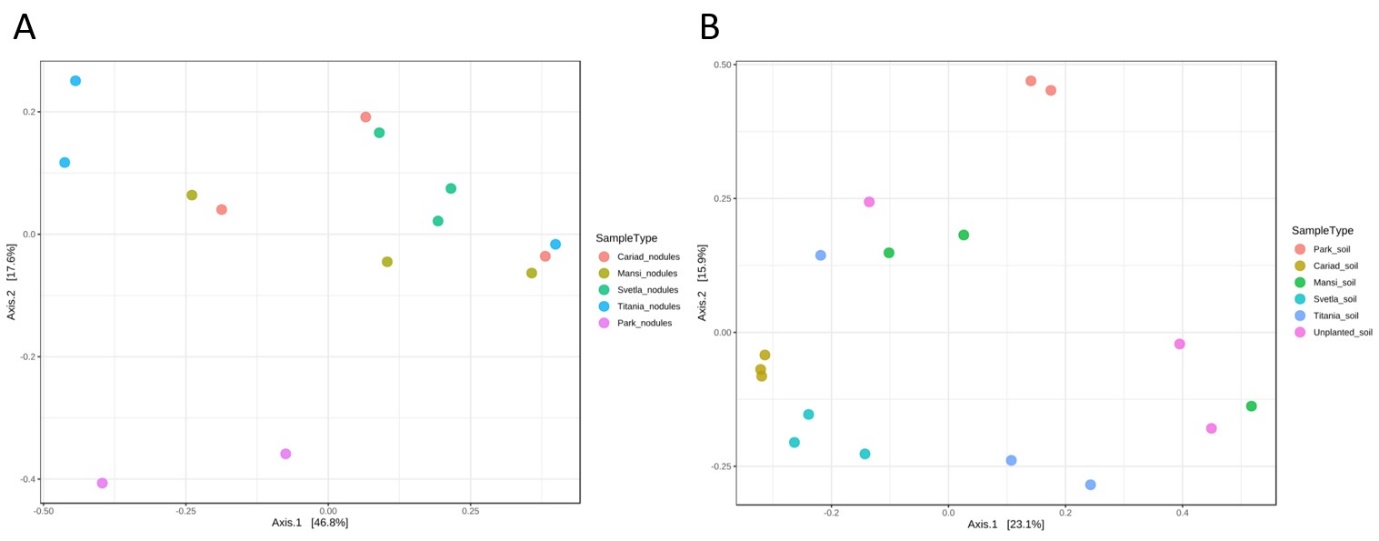
**Supplementary Figure S4**. A, Beta diversity of the nodule communities represented using two-dimensional principal component analysis calculated using Bray distance, [PERMANOVA] F-value: 1.3567; R-squared: 0.37616; p-value: 0.197; B, Beta diversity of the soil communities represented using two-dimensional principal component analysis calculated using Bray distance. [PERMANOVA] F-value: 1.9684; R-squared: 0.47221; p-value: 0.001.


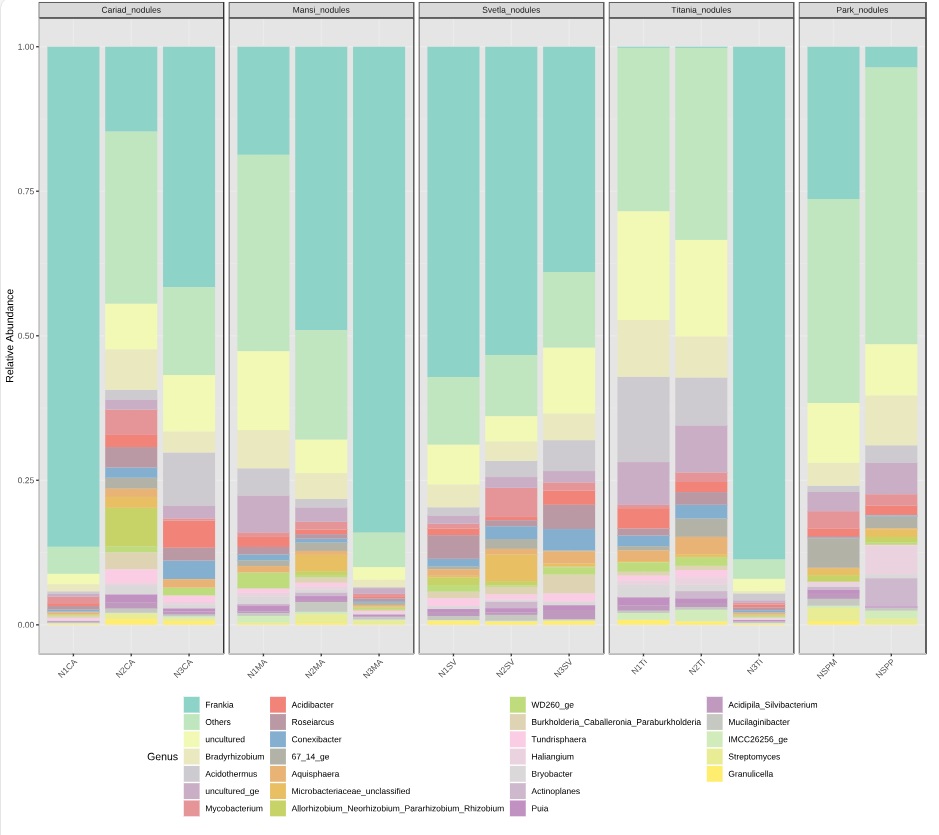
**Supplementary figure S5.** Percentage abundance of the top 25 most common genera for across the nodule communities. The Y-axis indicates the relative (%) abundance with the samples displayed on the X-axis, grouped by plots. Species outside the top 25 most abundant around grouped under the light green bar.


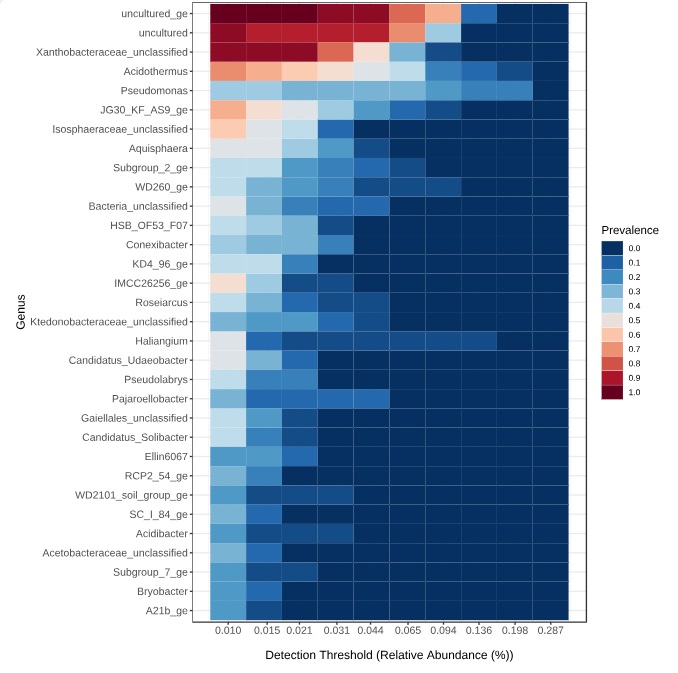
**Supplementary figure S6**. Core microbiome of soil samples, at the genus level represented using a heatmap. Red colouration indicates more prevalence and blue indicates less prevalence, relative abundance (%) is indicated along with X-axis.


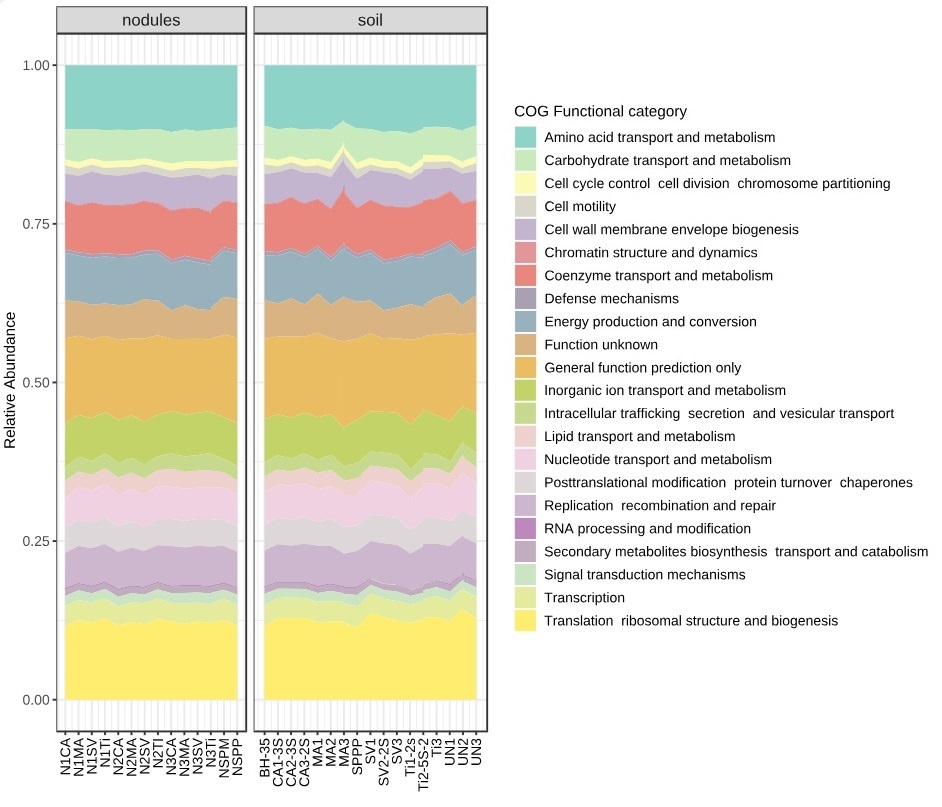
**Supplementary Figure S7.** Functional abundance profiling of the 16S community based upon predictions from PICRUSt^77-79^. The Y-axis indicates the relative (%) abundance with the samples displayed on the X-axis.
